# Extraction Optimization, Antioxidant, Cosmeceutical and Wound Healing Potential of *Echinacea purpurea* Glycerolic Extracts

**DOI:** 10.3390/molecules28031177

**Published:** 2023-01-25

**Authors:** Petar Ciganović, Lejsa Jakupović, Plamen Momchev, Laura Nižić Nodilo, Anita Hafner, Marijana Zovko Končić

**Affiliations:** 1 Faculty of Pharmacy and Biochemistry, University of Zagreb, A. Kovačića 1, 10000 Zagreb, Croatia; 2 Faculty of Pharmacy, Medical University-Sofia, Boulevard “Akademik Ivan Evstratiev Geshov” 15, 1000 Sofia, Bulgaria

**Keywords:** antioxidant, cosmeceutical, elastase, *Echinacea purpurea*, green extraction, tyrosinase, wound healing

## Abstract

*Echinacea purpurea* is a plant with immunomodulating properties, often used in topical preparations for treatment of small superficial wounds. In the presented study, the best conditions for ultrasound-assisted extraction of caffeic acid derivatives (caftaric and cichoric acid) (TPA-opt extract), as well as the conditions best suited for preparation of the extract with high radical scavenging activity (RSA-opt extract), from *E. purpurea* aerial parts were determined. A Box–Behnken design based on glycerol content (%, *w*/*w*), temperature (°C), ultrasonication power (W) and time (min) as independent variables was performed. Antioxidant, antiaging and wound healing effects of the two prepared extracts were evaluated. The results demonstrate that glycerol extraction is a fast and efficient method for preparation of the extracts with excellent radical scavenging, Fe^2+^ chelating and antioxidant abilities. Furthermore, the extracts demonstrated notable collagenase, elastase and tyrosinase inhibitory activity, indicating their antiaging properties. Well-pronounced hyaluronidase-inhibitory activities, with IC_50_ values lower than 30 μL extract/mL, as well as the ability to promote scratch closure in HaCaT keratinocyte monolayers, even in concentrations as low as 2.5 μL extract/mL (for RSA-opt), demonstrate promising wound healing effects of *E. purpurea.* The fact that the investigated extracts were prepared using glycerol, a non-toxic and environmentally friendly solvent, widely used in cosmetics, makes them suitable for direct use in specialized cosmeceutical formulations.

## 1. Introduction

The use of plants in topical preparations for medicinal and cosmetic applications is experiencing an unprecedented rise. Such products often display a broad spectrum of activities such as antiaging, antioxidant, anti-inflammatory, antipigmenting and many others. Such efficacy, which surpasses that of cosmetic products and more closely resembles the efficacy of pharmaceutical agents, led to the introduction of the popular new term “cosmeceutical”. Cosmeceutical is a topical preparation that is sold as a cosmetic product but has the performance characteristics that suggest a pharmaceutical action. As the term corresponds well with consumers’ expectations, it is often used in lay language even though it has no regulatory meaning [1]. In addition to displaying the desired activity and safety profile, modern cosmeceutical products should also have a satisfactory stability and sensory properties. Furthermore, as consumers are increasingly aware of the environmental impact, new, eco-friendly products are constantly being developed in order to meet such needs [2]. As plant-derived products originate from natural sources, they are in special demand in the cosmetic market, due to consumers’ perception of their extraordinary safety and bioactivity. It is widely considered that they can prevent and delay skin aging and deterioration. Indeed, numerous studies have shown that phenolics and other compounds, present in the plants and plant extracts, are desirable preservatives and functional ingredients in cosmetic products due to their antioxidant activity and their ability to impede numerous processes that negatively affect skin health and appearance [3].

One of the areas of cosmeceutical research is the design of green and sustainable extraction methods for bioactive natural products. For that purpose, the selection of an appropriate solvent is of utmost importance. Besides the high dissolving power, the ideal solvent should be safe, easy to handle and environmentally friendly [4,5]. One such solvent is glycerol, a natural, cost-efficient, non-toxic, biodegradable liquid with the additional benefit that it is manufactured from renewable sources, e.g., as a by-product of biodiesel production [6]. Furthermore, glycerol is one of the most widely used ingredients in cosmetic products, where it acts as humectant and viscosity-regulating agent [4]. Therefore, glycerol extracts of medicinal plants may have a dual role in cosmetic products, as active agents or excipients. Finally, the fact that glycerol may be incorporated into the final product makes glycerolic extraction highly attractive from the energy-saving point of view.

*Echinacea purpurea* (L.) Moench (Asteraceae) (purple coneflower) is a perennial medicinal herb with important immunostimulatory and anti-inflammatory properties. It is most frequently used for alleviation of common cold symptoms [7]. However, according to the European Medicines Agency, preparations of the aerial parts of *E. purpurea* are traditionally used for alleviation of skin disorders and minor wounds [8]. *E. purpurea* aerial parts contain diverse bioactive phytochemical constituents including essential oil, polysaccharides, phenolics, as well as nitrogen compounds, such as alkylamides, and small amounts of alkaloids. Among numerous constituents, caffeic acid derivatives and other phenolic acids are among the most prominent ones [9]. According to the European pharmacopoeial monograph for Echinaceae purpureae herba, caffeic acid derivatives are used for estimation of the quality of raw herbal material and its preparations [10].

The most abundant among caffeic acid derivatives in *E. purpurea* is cichoric acid, followed by caftaric acid. Cichoric acid displays a wide array of beneficial skin-related activities, such as antiviral, antioxidant and anti-inflammatory activity. In addition, cichoric acid may ameliorate inflammation induced by lipopolysaccharides (LPSs) in both cell culture and mice, as well as ameliorate UVA irradiation-induced dermal fibroblast senescence by inhibition of matrix metalloproteinase-3 activity. This opens the possibility of beneficial effects of cichoric acid on aging [11]. Caftaric acid may act as an antioxidant, anti-inflammatory, antimutagenic and anticarcinogenic agent, which adds to the beneficial effect on the skin [12]. In addition, a small dermatological study has shown that *E. purpurea* preparations may effectively improve the hydration of the skin and decrease skin wrinkling without inducing skin irritation [13].

Having in mind the traditional use and phytochemical composition of *E. purpurea*, the aim of this work was to optimize extraction of phenolic acids from *E. purpurea* aerial parts using glycerol, a non-toxic and eco-friendly solvent. Skin-related biological activities (antioxidant, enzyme inhibiting and wound healing effects) of the prepared extracts were investigated with the aim of obtaining highly active extracts suitable for use in cosmeceutical products.

## 2. Results and Discussion

### 2.1. Box–Behnken Design

Natural materials often contain a myriad of secondary metabolites among whom only a selected few have desirable pharmacological properties. Their amount in the extracts depends on their physicochemical properties, extraction solvent, type of extraction, as well as on numerous extraction parameters related to the type of extraction. Thus, finding the extraction procedure which yields the maximum amount of the target compound(s) may be a tedious and time-consuming procedure. In this work, efforts were undertaken to optimize ultrasound-assisted extraction (UAE) of caftaric and cichoric acids from *E. purpurea*. UAE is often used for extraction in solid/liquid systems because it is a simple, cost- and time-effective method, characterized by low CO_2_ emissions and solvent consumption.

Conditions for UAE were optimized using response surface methodology (RSM). It is a collection of mathematical and statistical techniques that enable building an empirical model between the response(s) of interest (dependent variables) and a number of associated independent variables. RSM is frequently applied for optimization of extraction of phenolic acids from various natural sources such as potatoes [14], birdsfoot trefoil [15] and *Rheum moorcroftianum* [16].

The results of a previous study have shown that, for the extraction of bioactive phenolic acids from *E. purpurea*, the concentration of glycerol used as solvent is of utmost importance. In that study, high glycerol content positively affected the extraction efficiency of phenolic acids [17]. Thus, for this study, glycerol concentrations higher than or equal to 50% (*w*/*w*) were used for the extraction. Furthermore, the temperature and the duration of the extraction, as well as the power of ultrasound, significantly affect the content of the target compounds. In order to fine-tune the extraction procedure and obtain the extracts with the maximum yields of phenolic acids and the most pronounced radical scavenging activity (RSA), RSM based on Box–Behnken design was used. The results are presented in Table 1. The extraction conditions greatly influenced the selected responses. For example, caftaric acid concentration varied greatly, from 13.07 µg/mL to 31.55 µg/mL in Run 4 and Run 5, respectively. Similarly, cichoric acid concentration spanned from 61.11 µg/mL to 103.26 µg/mL, again in Run 4 and Run 5, indicating that the same factors influence the extraction of both phenolic acids, which is expected due to their similar chemical structure. Similarly, the minimum and the maximum content of total phenolic acids (TPAs) was again reached in Run 4 and Run 5, respectively. Somewhat lower content of caffeic acid derivative in comparison with previous glycerol extraction of *E. purpurea* [17] may have contributed to the variability of plant material. RSA of the extracts varied greatly, from 8.17 µL extract/µL (Run 2) to 54.03 µL extract/µL (Run 18). While, expectedly, TPA correlated well with cichoric (*r*^2^ = 0.9969) and caftaric (*r*^2^ = 0.9773) acid content, no such correlation was found between TPA and RSA IC_50_. This means that, even though caffeic acid derivatives are strong antioxidants [18], other substances also contribute to the observed antiradical activity of the prepared extracts.

### 2.2. Model Analysis

Multiple regression analysis was used to analyze the experimental results. Table 2 shows the relationship between the independent and dependent variables in the form of polynomial equations. The contents of phenolic acids (caftaric acid, cichoric acid and TPA) were influenced by all the selected independent variables as quadratic terms. Glycerol content and temperature were preceded with positive coefficients, while negative coefficients preceded the USP and time. This means that the extreme values of glycerol content and temperature negatively affect the content of phenolic acids, while the opposite is true for USP and time. Additionally, glycerol content and temperature influenced the phenolic acid content as negative and positive linear terms, respectively. Interestingly, high glycerol content positively affected the extraction efficiency of phenolic acids in an earlier study [17], while glycerol’s influence was negative in this study. This apparent discrepancy is due to difference in lowest and highest glycerol contents. Namely, the first study used a much wider range of glycerol content (10–90%, *w*/*w*) and only the highest concentrations from that study (50–90%, *w*/*w*) were those selected for this investigation. On the other hand, independent variables influenced the antiradical activity’s square root as linear terms preceded with either a positive (glycerol content) or negative (temperature and time) coefficient. This means that relatively high glycerol content, as well as lower temperature and extraction time, will produce extracts with high RSA IC_50_ and consequently low RSA activity.

As demonstrated by ANOVA (Table 3), the relationship between the response variables and independent variables is satisfactorily expressed using the polynomial equations presented in Table 2. The statistical significance of each model was calculated using the *F*-test and *p*-values. The calculated *F*-values were higher than 5, while the *p*-values were 0.002 or lower. This indicates that the models are highly significant and that they can be used to optimize the extraction variables. Lack-of-fit in the models was statistically insignificant relative to the pure error which demonstrated that the fitting model is adequate to describe the experimental data. The determination coefficients (*R*^2^) for phenolic acid content were approaching 0.9, showing that the observed values are well replicated by the model. However, in the case of antiradical activity, *R*^2^ was rather low (0.4928). The predicted *R*^2^ were in reasonable agreement with the adjusted ones, further confirming that the models may be used to predict and optimize the amount of target substances in the extracts.

### 2.3. Validation of Optimal Extraction Conditions

**Figure 1:**
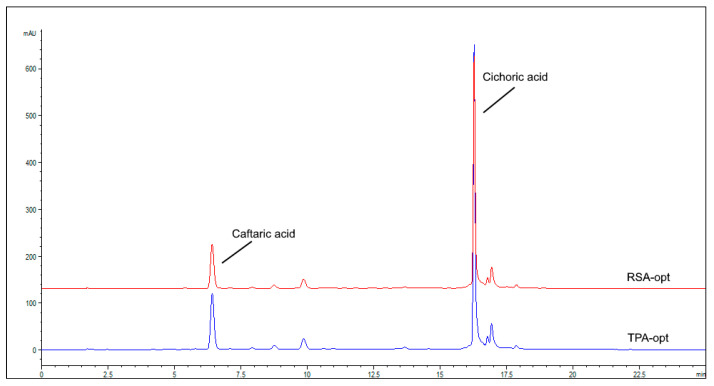
Chromatograms of RSA-opt and TPA-opt recorded at 330 nm.

Based on the experimental results and statistical analysis, numerical optimizations were conducted to establish the optimum levels of independent variables (Table 4). As previously mentioned, the most important extraction factor for all the investigated parameters was glycerol concentration. It is well known that the extraction solvent greatly affects the extraction efficiency. In this work, the glycerol content needed for optimal extraction of specific phenolic compounds varied according to the response. In general, phenolic acids were best extracted using moderate glycerol concentration as reflected in the maximized TPA at 70%. The values for extraction temperature, USP and time were approaching the maximum values used in the Box–Behnken design, indicating their relatively good stability in the extraction medium. The best antiradical activity, on the other hand, was achieved using low glycerol concentration and lower ultrasonication power, indicating that, in addition to phenolic acids, other compounds of relatively lower polarity and higher sensitivity are partly responsible for the observed antiradical effects. The predicted results matched well with the experimental ones, with relatively low deviations from calculated values, indicating good suitability of the selected models (Table 4). The HPLC-DAD chromatograms of the two prepared extracts are shown in Figure 1.

### 2.4. Antioxidant Activity of the Optimized Extracts

Botanical ingredients represent one of the largest categories of natural active substances used in dermatology. In order to investigate *E. purpurea* extracts as potentially valuable cosmeceutical ingredients, their biological activity was determined using several methods. Antioxidant activity of cosmetic product ingredients is of utmost importance because they may act both as preservatives and active components in cosmeceutical products. Antioxidants may protect the cosmetic product against the oxidation that occurs during its storage and use by scavenging free radicals [19]. Chelation of metal, such as pro-oxidant Fe^2+^ and other ions, is also very important because they may induce peroxidation of polyunsaturated fatty acids that natural cosmetics are especially rich in [20]. Finally, functional cosmeceutical ingredients may have a more active role in such products. They offer protection against oxidative damage of skin macromolecules associated with the effects of free radicals and UV radiation on the skin [21,22]. Thus, in this work the influence of the prepared extracts on the free radicals (as modeled by DPPH free radical), chelating activity on Fe^2+^ ions and the activity in heat-induced unsaturated fatty acid degradation in a β-carotene–linoleic acid system were investigated and compared with the activity of standard antioxidants, butylated hydroxyanisole (BHA) and ethylenediaminetetraacetic acid (EDTA). Even though the activity of the extracts may not be directly compared to the activity of standard antioxidants, due to the fact that they are expressed in different measurements units (the activity of the extracts and standards was expressed as μL/mL and μg/mL, respectively), it is possible to regard the activity of the standards as volume equivalents of 1 mg/mL solutions. Thus, it was reported for general comparison purposes.

**Figure 2:**
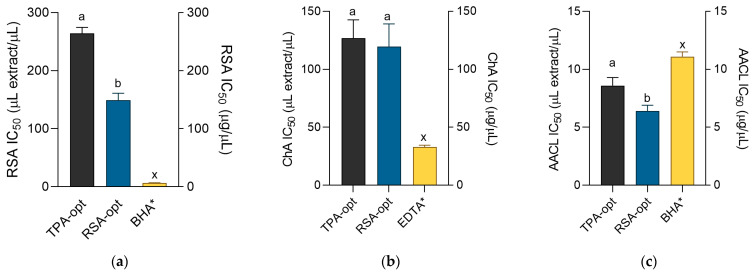
Antiradical activity (**a**), chelating activity (**b**) and the activity in β-carotene–linoleic acid assay (**c**) of the extracts and positive controls BHA (butylated hydroxyanisole) and EDTA (ethylenediaminetetraacetic acid). ^a,b^ = Differences between the extracts within a column (*t*-test, *p* < 0.05). ^x^ = differences from the positive control (Dunnett’s post-test, *p* < 0.05). Columns not sharing the same letter are statistically different. Asterisk indicates that the unit is placed at the right ordinate.

Figure 2a–c depict the results of the antioxidant assays performed in this work. Antiradical and chelating activities of the extracts were lower than the activity of the standards solutions. However, in the β-carotene–linoleic acid assay, the extracts were notably stronger antioxidants than BHA. The activity of the individual extracts differed according to the assay. The prepared optimized extracts were similarly efficient Fe^2+^ ion chelators with IC_50_ values of approximately 120 μL of extract per mL of solution. However, expectedly, RSA-opt was a stronger radical scavenger than TPA-opt. Both extracts inhibited thermally induced degradation of the β-carotene–linoleic acid system. Since this assay is based on the ability of the mixture components to react with linoleic radical and other radicals formed in the solution, the high activity of RSA-opt, the extract optimized to display pronounced antiradical activity, is not surprising. Caffeic acid and its derivatives are strong antioxidants. For example, in many antioxidant assays caffeic acid shows activity that often surpasses the activity of standard antioxidants, ascorbic acid and trolox. Additional advantages of caffeic acid include higher stability than ascorbic acid and, unlike trolox, the possibility of extraction from natural sources [18]. Furthermore, cichoric acid, the main caffeic acid derivatives in *E. purpurea* extracts [23], as well as caftaric acid [12] also display potent antioxidant activity. However, it seems that caffeic acid derivatives are not the only antioxidant molecules in *E. purpurea* because RSA-opt, the most active radical-scavenger, contained lower amounts of caffeic derivatives than TPA-opt.

### 2.5. Cosmeceutical Activity of the Optimized Extracts

In addition to hydration and antioxidant protection, contemporary cosmetic products are expected to have additional properties that beneficially affect skin appearance. Excessive enzymatic activity in the skin, caused by environmental factors and aging, can cause premature breakdown of skin proteins, such as elastin or collagen, as well as breakdown of polysaccharides, such as hyaluronic acid. By inhibiting the enzymes responsible for degradation of skin macromolecules, plant metabolites may decelerate the skin aging process and reduce its aesthetically visible effects, such as dehydrated skin, reduced elasticity, dark spots and the appearance of wrinkles [24].

**Figure 3:**
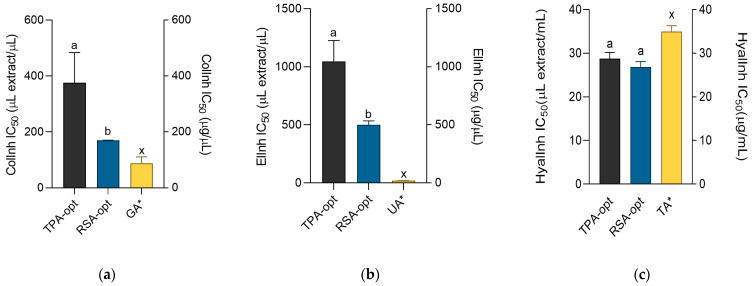
Collagenase (**a**), elastase (**b**) and hyaluronidase (**c**) inhibitory activity of the extracts and positive controls gallic acid (GA), ursolic acid (UA) and tannic acid (TA). ^a,b^ = Differences between the extracts within a column (*t*-test, *p* < 0.05). ^x^ = Differences from the positive control (Dunnett’s post-test, *p* < 0.05). Columns not sharing the same letter are statistically different. Asterisk indicates that the unit is placed at the right ordinate.

Skin proteins play a pivotal role in maintaining not only the function and form, but also youthful appearance, of the skin. Fibrillar collagen is the most abundant skin protein that constitute three-quarters of skin’s dry weight, while the amount of elastin fibers is substantially lower. Collagen is responsible for the strength and stability of skin tissue because sliding and realignment of collagen fibrils allows skin to deform while maintaining its integrity and preventing damage. On the other hand, elastin fibers contribute extensibility and reversible recoil to skin, which allows for skin to return to its resting state after external force is removed [25]. Collagenase is the enzyme active in the extracellular matrix that catalyzes degradation of collagen. As a reaction to aging or external influences (e.g., UV radiation), its activity increases. This leads to the formation of wrinkles and loss of skin tone [26]. Degradation of elastin is induced by the enzyme elastase, which is directly related to skin aging and oxidative stress [27]. Clinical trials confirm that natural products and other compounds that display inhibition of elastase have significant antiaging potential [28]. The collagenase- and elastase-inhibitory effects of the extracts are shown in Figure 3a,b. Even though the extracts were weaker collagenase and elastase inhibitors than the 1 mg/mL solutions of positive controls, gallic and ursolic acid, respectively, they still showed a significant degree of inhibition of these two enzymes. In both assays, RSA-opt was the more active extract. Previously, it was found that aqueous *E. purpurea* extract was a potent collagenase and elastase agent [29]. In addition, grape pomace extracts, rich in caftaric acid and other phenolic acids, showed inhibitory effects on both collagenase and elastase enzyme activities [30], indicating contribution of this phenolic acid to the observed activity of the extracts.

Reduced hydration of skin is characterized by a reduced turgor, resilience and elasticity and loss of youthful appearance. Hyaluronic acid is a polysaccharide found in human skin that possesses extreme water retaining capacity. As such, it is one of the most important molecules responsible for skin hydration [31]. In various pathological processes, as well as during physiological skin aging, hyaluronic acid is increasingly degraded by hyaluronidase, the enzyme that controls the turnover of hyaluronic acid [32]. Thus, inhibition of hyaluronidase leads to retention of skin moisture and is one of the most promising approaches for the prevention of premature skin aging. As presented in Figure 3c, both extracts were excellent hyaluronidase inhibitors, with the activity surpassing that of the positive control, tannic acid. This is in line with a previous observation that aqueous *E. purpurea* extract possessed a significant antihyaluronidase activity [29]. This activity may be mediated by caffeic acid derivatives present in the extracts. Previous research shows that chicoric and caftaric acid have excellent antihyaluronidase activity [33]. Furthermore, chicoric acid, the main ingredient of TPA-opt, was found to inhibit human hyaluronidase 1, the enzyme that degrades high molecular weight hyaluronic acid [34]. Other caffeic acid derivatives may also add to the beneficial effect on wound healing. For example, echinacoside displays antihyaluronidase properties [35] and thus contributes to the observed effects of *E. purpurea* extracts.

**Figure 4:**
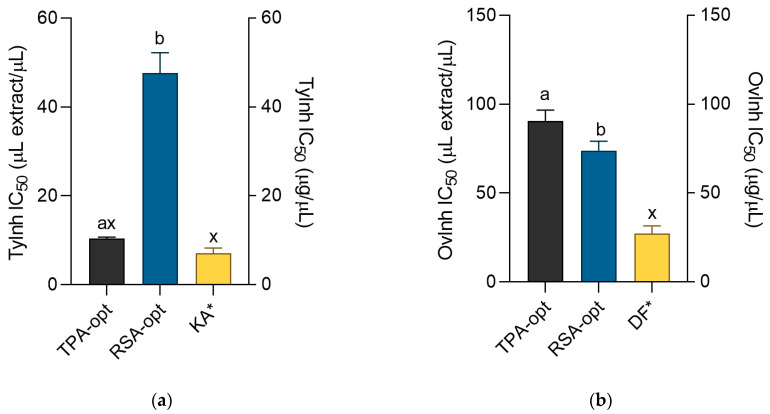
Tyrosinase inhibiting (**a**) and anti-inflammatory (**b**) activity of the extracts and positive controls kojic acid (KA) and diclofenac (DF). ^a,b^ = Differences between the extracts within a column (*t*-test, *p* < 0.05). ^x^ = Differences from the positive control (Dunnett’s post-test, *p* < 0.05). Columns not sharing the same letter are statistically different. Asterisk indicates that the unit is placed at the right ordinate.

Damage caused by UV radiation may be prevented by melanin, a photoprotective macromolecular pigment synthetized in the epidermis, with the enzyme tyrosinase catalyzing the first, rate-determining step. Most of the time, production of melanin is a beneficial or welcomed physiological reaction. However, in some cases, such as aging or melasma, irregularly distributed production of melanin results in uneven skin pigmentation and represents an esthetic problem for the affected individual. As tyrosinase inhibitors block melanogenesis and prevent hyperpigmentation of the skin, their inclusion in cosmetic products is desirable from an aesthetic point of view [36]. Although both investigated extracts showed notable antityrosinase activity (Figure 4a), the effectiveness of the TPA-opt extract was much more pronounced and statistically equal to the activity of the standard, kojic acid. Caftaric acid, present in TPA-opt, was shown to be a competitive tyrosinase inhibitor, and proposed as a promising ingredient in cosmetic products with skin whitening properties [37].

Denaturation of tissue proteins is one of the characteristics and causes of inflammatory processes in the body [38]. Therefore, the suppression of protein denaturation may impede the development of inflammatory skin changes which is another important aspect of antiaging activity [39]. Although all the investigated extracts were able to inhibit heat-induced ovalbumin coagulation (Figure 4b), better anti-inflammatory activity was displayed by RSA-opt. Caftaric acid may be partly responsible for the observed effect because it was previously demonstrated that it acts as an anti-inflammatory agent [12]. It is important to say that glycerol probably plays a crucial role in this assay. Namely, the OVInh IC_50_ of glycerol in this assay was 19.55 ± 0.01 μL/mL, indicating that most of the observed activity in this assay is due to the presence of glycerol in the extracts. The role of glycerol as an active solvent that prevents the denaturation of proteins such as collagen has been previously established [40]. This experiment further confirms that the benefits of glycerol for the preparation of cosmeceutical extracts extend beyond its application as a green extraction solvent.

### 2.6. Evaluation of Cell Viability

In order to determine not only the toxicity of the prepared extracts, but also the concentration range in which the wound healing assay should be conducted, the influence of the prepared *E. purpurea* glycerol extracts and the solvents used for their preparation (glycerol in the appropriate dilutions) on cell viability was tested. An experiment was performed using the 3-(4,5-dimethylthiazol-2-yl)-2,5-diphenyltetrazolium bromide test (MTT test) on HaCaT cells, a long-lived, spontaneously immortalized human keratinocyte line, able to differentiate in vitro [41]. HaCaT cells are often used as a suitable model for testing of wound healing activity [42]. Different concentrations (2.5–250 μL/mL) of the extracts and corresponding solvents, diluted in Hanks’ balanced salt solution (HBSS), were used to estimate the toxicity of the extracts and solvents to HaCaT cell cultures.

**Figure 5:**
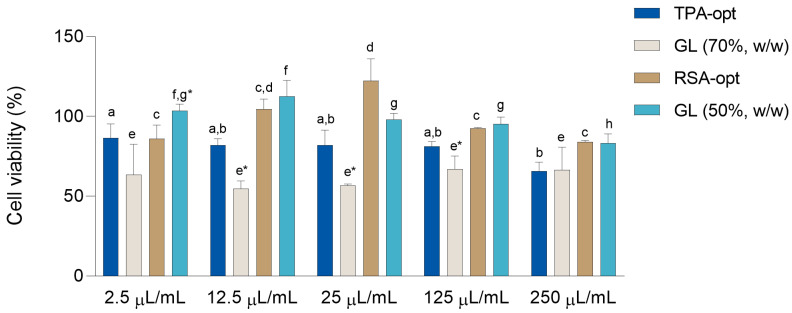
The influence of different extracts and glycerol (GL) dilutions on the survival of HaCaT cells. Cell survival is expressed as a percentage when compared to cells treated with HBSS. The results are shown as the mean ± SD (n = 3). ^a,b^ = Differences between the different dilutions of TP-opt extracts. ^c,d^ = Differences between the different dilutions of RSA-opt extracts. ^e^ = Differences between the different dilutions of 70% (*w*/*w*) glycerol. ^f–h^ = Differences between the different dilutions of 50% (*w*/*w*) glycerol (one-way ANOVA followed by Tukey’s post-test, *p* < 0.05). Columns prepared from the same concentration of extract and glycerol not sharing the same letter are statistically different. * = Difference between the extract and the corresponding solvent (paired *t*-test, *p* < 0.05).

The results are presented in Figure 5. Except for the difference between the highest and the lowest concentration of the extract, the viability of the cells treated with TPA-opt did not differ across concentrations. Among the RSA-opt dilutions, the cells treated with 25 μL/mL concentration showed the highest viability. Differences in dilution of the 70% (*w*/*w*) glycerol did not significantly affect viability. Among the different dilutions of 50% (*w*/*w*) glycerol, lower viability was recorded in the cells treated with the highest concentrations (one-way ANOVA followed by Tukey’s post-test, *p* < 0.05, comparisons of different dilutions of the same extract/glycerol concentration). Relatively high viability of the cells treated with most extracts and solvent dilutions, recorded in this assay, indicates their low toxicity. The comparison of equal concentrations of the extracts and the solvents used for their preparation shows that the cells treated with the same concentrations of RSA-opt and 50% (*w*/*w*) glycerol most often show no statistically significant difference in their viability. On the other hand, HaCaT cells treated 70% (*w*/*w*) glycerol generally showed significantly lower viability than those treated with TPA-opt (paired *t*-test, *p* < 0.05). This could indicate the protective effect of *E. purpurea* extract and the phytochemicals contained in it. Based on the results of the MTT assay, 10 samples were tested for their wound healing activity: TPA-opt and 70% (*w*/*w*) glycerol (in concentrations 2.5 μL/mL and 12.5 μL/mL), as well as RSA-opt and 50% (*w*/*w*) glycerol (in concentrations 2.5 μL/mL, 12.5 μL/mL and 25 μL/mL).

### 2.7. Wound Healing Effects of E. purporea

Wound healing is a process of dynamic cellular and molecular mechanisms, divided into several stages, which may overlap over time: hemostasis, inflammation, proliferation/migration and maturation or remodeling, characterized by the formation of new tissue. In the proliferation phase, the migration of keratinocytes and fibroblasts recovers the network of blood vessels and participates in the granulation process. This characteristic is used for the in vitro “scratch” test method. In this procedure, a scratch that leaves an empty space (“wound”) on the well bottom is created in a cell monolayer. If the conditions are satisfactory, cell movement and proliferation occur, followed by the gradual closure of the cell model wound [43].

**Figure 6:**
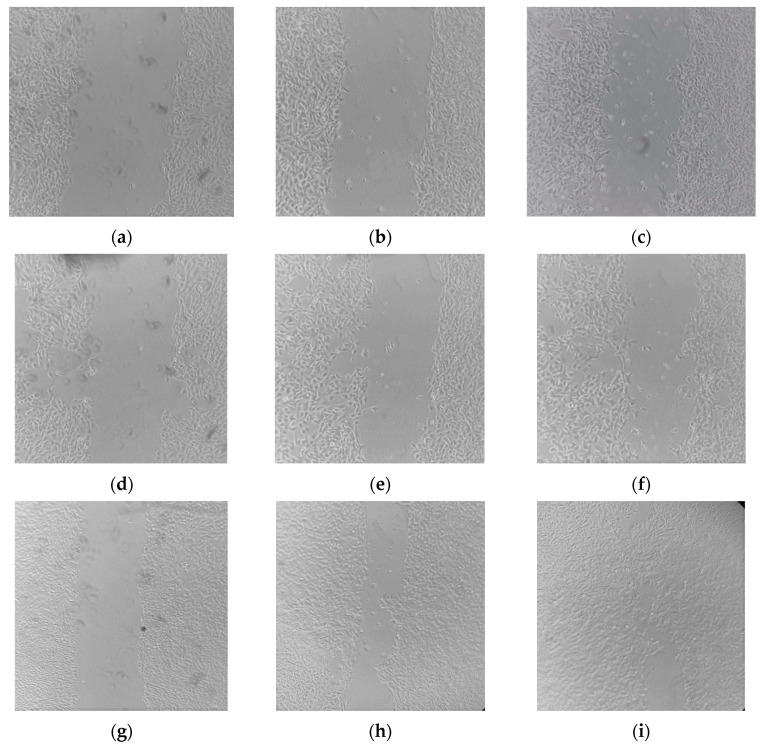
The influence of the HBSS (**a**–**c**), TPA-opt (**d**,**e**) and RSA-opt (**g**–**i**) in 2.5 μL extract/mL dilutions on the closure of scratch in HaCaT cell monolayer after 0 h (**a**,**d**,**g**), 24h (**b**,**e**,**h**) and 48 h (**c**,**f**,**i**) after being incubated with the extracts or HBSS for 2 h.

In our research, cells were treated with different dilutions of *E. purpurea* extracts and glycerol. HBSS was used as a negative control. The wound-closure process was followed over 48 h. Figure 6 depicts the closure of the wounds treated with different samples. For this depiction, the extracts were used in a 2.5 μL/mL concentration and quantitatively compared with HBSS. Both extracts accelerated wound closure in a confluent cell layer (Figure 6a–c). The model wounds of the HaCaT cells treated with the extracts tended to be reduced over time. The RSA-opt sample was especially active. After 48 h, the scratch surface in the cell monolayer treated with that extract was barely visible, indicating excellent wound healing activity. On the other hand, the reduction of the wound surface in cells treated with the negative control was barely noticeable.

**Figure 7:**
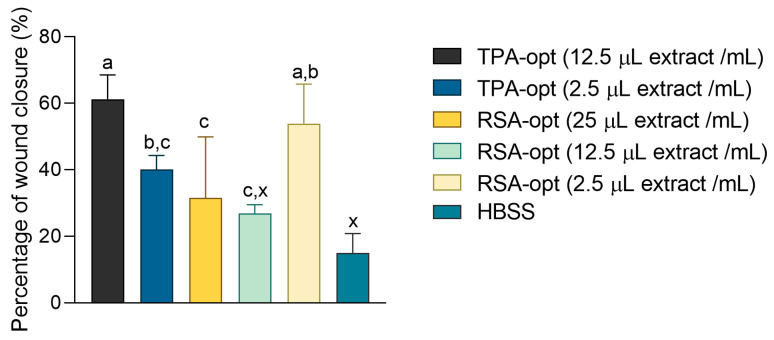
The influence of the different extract dilutions on 48 h wound closure in HaCaT cells. ^a–c^ = Differences between the extracts (one-way ANOVA followed by Tukey’s post-test, *p* < 0.05). ^x^ = Differences from the negative control (one-way ANOVA followed by Dunnett’s post-test, *p* < 0.05). Columns not sharing the same letter are statistically different.

Figure 7 presents the percentage of wound closure (percentage of wound surface reduction relative to the wound surface at the beginning of the treatment at 0 h) after 48 h. The activity of the different glycerol dilutions was also tested, but since their activity was equal to or even lower than the activity of the HBSS control, they were omitted from the figure and the subsequent analysis. The lack of solvent activity also indicates that the phytochemical constituents were responsible for promotion of the proliferation of HaCaT cells during the tested incubation time. Interestingly, the wound healing activity of RSA-opt was not dose-dependent. For example, RSA-opt in a concentration of 2.5 μL extract/mL showed better wound healing activity than in a concentration of 12.5 μL extract/mL. Concentration-independent wound healing activity of herbal extracts is an occurrence that is not uncommon. The reason may be a complex interplay between the extracts’ components, both those that accelerate wound healing and those that oppose it. Thus, one of the future research directions may be focused towards finding the components that are primarily responsible for the observed wound healing activity of the *E. purpurea* extracts, as well as the optimal dose range for the application of the extracts. Similar behavior of plant-based preparations has also been recorded in vivo, e.g., with ointment containing *Ocimum gratissimum* leaf extract [44]. The best activity was recorded in TPA-opt and RSA-opt extracts in the concentration of 12.5 μL extract/mL and 2.5 μL extract/mL, respectively. The activity among the other tested extracts and concentrations did not statistically differ (one-way ANOVA followed by Tukey’s post-test, *p* < 0.05). This confirms the traditional indication of the European Medicines Agency, that *E. purpurea* and preparations thereof may be used in herbal medicinal products for alleviation of skin disorders and minor wounds [8].

## 3. Materials and Methods

### 3.1. Chemicals

Butylated hydroxyanisole (BHA, ≥98.5%), chlorogenic acid (European Pharmacopoeia Reference Standard), diclofenac (≥98%), kojic acid (≥98.5%), (3-[4,5-dimethylthiazol-2-yl]-2,5-diphenyltetrazolium bromide, collagenase from *Clostridium histolyticum*, hyaluronidase from bovine testes, mushroom tyrosinase and porcine pancreas elastase were purchased from Sigma-Aldrich (St. Louis, MO, USA). Soybean LOX was a product from TCI chemicals (Tokyo, Japan). Acetonitrile was HPLC grade. Other reagents and chemicals were of analytical grade.

### 3.2. Plant Material

The commercially available *E. purpurea* aerial parts, consisting of leaves, stalks and flowers, were supplied by the company Suban. The identity was confirmed by the authors (P.C. and M.Z.K.) using the Echinaceae purpureae herba monograph of the European Pharmacopoeia [10]. Plant material was milled and passed through a sieve of 850 μm mesh size. A voucher specimen (EP-2021) was deposited in the Department of Pharmacognosy, Faculty of Pharmacy and Biochemistry, University of Zagreb.

### 3.3. Preparation of the Extracts According to Box–Behnken Design

For the Box–Behnken design, the following independent variables were used: glycerol concentration of 50–90%, *w*/*w*, temperature of 40–70 °C, ultrasound power of 72–360 W and time of 20–60 min. Powdered plant material (0.1 g) was suspended in 30 mL of a glycerol/water mixture in a 50 mL Erlenmeyer flask. The extraction was performed in an ultrasonic bath using frequency of 35 Hz at various temperatures, ultrasonication strengths and time intervals. The details are presented in Table 1. Upon the extraction, the mixtures were filtered and stored in the dark at −20 °C until analysis.

### 3.4. HPLC Determinations of Caffeic Acid Derivatives

The content of caffeic acid derivatives was determined according to the method described in the monograph of Echinaceae purpureae herba in the European Pharmacopoeia [10]. For the analysis, an Agilent 1200 series (Agilent Technologies, Santa Clara, CA, USA) equipped with an autosampler and a DAD detector was used. Separation was performed on a Zorbax Eclipse XDB-C18 column (5 µm, 12.5 mm × 4.6 mm, Agilent, Santa Clara, CA, USA). The prepared extracts and the standard (0.025 mg/mL chlorogenic acid in 70% ethanol) were filtered through a PTFE syringe filter with pore size of 0.45 μm. Mobile phase A (phosphoric acid and water, 1:999 V/V) and mobile phase B (acetonitrile) were used according to the following protocol: 0-13 min (90–78% A), 13–14 min (78–60% A), 14-20 min (60–40% A). The analysis was performed at 35 °C using flow rate of 1.5 mL/min, and the chromatograms were recorded at 330 nm. TPA was calculated as the sum of caftaric and cichoric acid content.

### 3.5. Radical Scavenging Activity

Radical scavenging activity (RSA) was assessed using the 2,2-diphenyl-1-picrylhydrazyl (DPPH) free radical [45] method. To 130 μL of the extract or BHA (1 mg/mL) solution in methanol, 70 μL of DPPH (0.21 mg/mL) solution was added. After 30 min of incubation at room temperature, the absorbance was recorded at 545 nm. RSA was calculated according to Equation (1):

(1) $$\mathrm{RSA}\;\left(\%\right)=\frac{A_{0}-A_{s}}{A_{0}}\times 100$$

where A_0_ is the absorbance of the negative control which used methanol instead of the extract and A_s_ is the absorbance of the respective extract. Concentration of the extract which scavenged 50% of free radicals present in the solution (RSA IC_50_) was calculated.

### 3.6. Fe^2+^ Chelating Activity

The chelating activity (ChA) was studied as described in [46]. To the solution of the extract in methanol (150 μL), 0.25 mM FeCl_2_ solution (50 μL) was added. After 5 min of incubation, ferrozine solution was added (1.0 mM, 100 μL). Absorbance at 545 nm was recorded after 10 min. ChA was calculated using Equation (2):

(2) $$\mathrm{ChA}\;\left(\%\right)=\frac{A_{0}-A_{s}}{A_{0}}\times 100$$

where A_0_ is the absorbance of the negative control (which used methanol instead of the extract) and A_s_ is the absorbance of the respective extract. Concentration of the extract which chelates 50% of Fe^2+^ present in the solution (ChA IC_50_) was calculated. EDTA (1 mg/mL) was used as positive control.

### 3.7. Antioxidant Activity in β-Carotene–Linoleic Acid Assay

The activity was evaluated according to a modified literature procedure [47]. The extract solution in methanol (50 μL) was added to 200 μL of emulsion containing β-carotene (6.7 μg/mL), linoleic acid (0.7 mg/mL) and Tween 40 (6.7 mg/mL). The reaction mixture was incubated at 50 °C. The antioxidant activity in the β-carotene–linoleic acid assay (AACL) was calculated based on the absorbances recorded after 60 min using Equation (3):

(3) $$\mathrm{AACL}\;\left(\%\right)=\frac{A_{\mathrm{sample}}}{A_{\mathrm{control}}}\times 100$$

where A_control_ and A_sample_ are the absorbances of the methanol control and the extract, respectively. Concentration of the extract that protects 50% of β-carotene present in the solution (AACL IC_50_) was calculated. BHA (1 mg/mL) was used as positive control.

### 3.8. Collagenase Inhibitory Activity

In 50 mL of citrate buffer (0.2 M, pH 5.0), 80 mg of SnCl_2_ × 2H_2_O was dissolved [48]. Ninhydrin solution was prepared by dissolving 0.5 g of ninhydrin in 10 mL of DMSO. The ninhydrin reagent for color development was made by mixing SnCl_2_ solution with an equal volume of ninhydrin solution before use. To the solution of the extract, gelatin (7 µL, 2 mg/mL) and collagenase (7 µL, 1 mg/mL) were dissolved in reaction buffer (50 mM Tris-HCl, pH 7.5, 5 mM CaCl_2_ and 1 µM ZnCl_2_). Quench buffer contained 12% (w/v) PEG 6000 and 25 mM EDTA. The inhibition of collagenase (ColInh) was calculated by using the following Equation (4):

(4) $$\mathrm{ColInh}\;\left(\%\right)=\frac{A_{0}-A_{s}}{A_{0}}\times 100$$

where A_0_ is the absorbance of the negative control (water) and A_s_ is the absorbance of the respective extract. Concentration of the extract which inhibits 50% of the ovalbumin coagulation (ColInh IC_50_) was calculated. Gallic acid (1 mg/mL) was used as the positive control.

### 3.9. Elastase Inhibitory Activity

Elastase inhibitory activity was determined as described previously [49]. To the 100 μL of extract solution in Tris-HCl buffer (0.1 M, pH 8.0), 1 mM N-succinyl-(Ala)_3_-nitroanilide in the same buffer was added. Elastase solution was added after 10 min and the absorbance was measured at 410 nm after an additional 10 min. Elastase inhibitory activity (ElInh) was calculated as follows (Equation (5)):

(5) $$\mathrm{ElInh}\;\left(\%\right)=\frac{A_{0}-A_{s}}{A_{0}}\times 100$$

where A_0_ is the absorbance of the negative control (solution where instead of extract the Tris-HCL buffer was used) and A_s_ is the absorbance of the respective extract. Ursolic acid (1 mg/mL) was used as the standard elastase inhibitor.

### 3.10. Hyaluronidase Inhibitory Activity

For hyaluronidase (LOX) inhibitory activity [50], 25 µL of the extract solution and 20 µL of hyaluronidase solution (4 mg/mL) were mixed and incubated for 20 min at 37 °C. After 20 min, 40 µL of 12.5 mM CaCl_2_ was added and incubated for an additional 20 min at 37 °C. Sodium hyaluronate (50 µL, 3.5 mg/mL) was added and incubated for at 37 °C with constant shaking. After 40 min, the reaction was stopped by adding 20 µL of 0.9 M NaOH and 40 µL of 0.2 M sodium tetraborate and heating for 3 min at 100 °C. Then, 160 µL of *p*-dimethylaminobenzaldehide reagent (DMABA) (0.25 g DMABA dissolved in 4.4 mL of acetic acid and 0.6 mL of 10 M HCl) was added and the reaction mixture was incubated at 37 °C for an additional 10 min. Absorbance was measured at 585 nm. Tannic acid was used as positive control. Hyaluronidase inhibitory activity (HyalInh) was calculated as shown in Equation (6):

(6) $$\mathrm{HyalInh}\;\left(\%\right)=\frac{A_{0}-A_{s}}{A_{0}}\times 100$$

where A_0_ is the absorbance of the negative control and A_s_ is the absorbance of the corresponding extract. HyalInh IC_50_ was calculated as the concentration of the extract that inhibited 50% of hyaluronidase activity and is expressed as μL of extract/mL of solution.

### 3.11. Tyrosinase Inhibitory Activity

The activity was determined following the method described in [49]. To the extract solution (80 μL), 40 μL of tyrosinase solution (in 16 mM pH 6.8 phosphate buffer) was added. After 10 min in the dark at 25 °C, 80 μL of L-DOPA solution (0.19 mg/mL in phosphate buffer) was added. The absorbance was measured at 492 nm after 10 min. Tyrosinase inhibitory activity (TyInh) was calculated as (Equation (7))

(7) $$\mathrm{TyInh}\;\left(\%\right)=\frac{A_{0}-A_{s}}{A_{0}}\times 100$$

where A_0_ is the absorbance of the negative control (where buffer was used instead of the extract) and A_s_ is the absorbance of the respective extract. Concentration of the extract which inhibits 50% of tyrosinase activity (TyInh IC_50_) was calculated. Kojic acid (1 mg/mL) was used as positive control.

### 3.12. Inhibition of Heat-Induced Ovalbumin Coagulation

The activity was evaluated by the heat-induced ovalbumin coagulation method [39]. To 0.4 mL of fresh ovalbumin solution, 2.8 mL of phosphate buffered saline (pH 6.4) and 2 mL of the extract solution were added. After 15 min at 37 °C, the solutions were heated at 70 °C for 5 min. Upon cooling of the reaction mixture, the absorbance was recorded at 660 nm. The inhibition of denaturation (OvInh) was calculated by using the following Equation (8):

(8) $$\mathrm{OvInh}\;\left(\%\right)=\frac{A_{0}-A_{s}}{A_{0}}\times 100$$

where A_0_ is the absorbance of the negative control (water) and A_s_ is the absorbance of the respective extract. Concentration of the extract which inhibits 50% of the ovalbumin coagulation (OvInh IC_50_) was calculated. Diclofenac sodium (1 mg/mL) was used as the positive control.

### 3.13. Cell Culture Conditions

The HaCaT human keratinocyte cell line (CLS Cell Line Services, Heidelberg, Germany) was cultivated using Dulbecco’s modified Eagle medium (DMEM) (St. Louis, MO, USA) supplemented with fetal bovine serum (10%, Biosera, Boussens, France), penicillin, streptomycin and amphotericin B (5%, Lonza, Basel, Switzerland). The cells were passaged at 80–90% confluence. The medium was changed approximately every 48 h. The cultures were maintained at 95% humidity and 37 °C in an atmosphere of 5% CO_2_.

### 3.14. Cell Viability Study

Cell viability was determined with the colorimetric MTT assay. HaCaT cells were seeded onto 96-well plates at a density of 2 × 10^4^ cells/well and allowed to reach confluence over 24 h. Solutions of the extracts were mixed with Hank’s balanced salt solution (HBSS; pH 6.0, Capricorn Scientific, Ebsdorfergrund, Germany). Prior to the treatment with the extracts, the cell culture medium was withdrawn, and the cells washed with HBSS. The cells were then exposed to the solutions of the extracts in concentrations of 2.5–250 μL/mL for 2 h. Cells incubated in HBSS were used as a negative control. After 2 h of treatment with the extracts, the cells were washed twice with HBSS and incubated with fresh medium (500 µL/well) for 24 h. A total of 50 µL of the MTT solution (5 mg/mL) was added to each well. After 1 h at 37 °C, the medium was removed, and the cells were lysed. Formazan was dissolved with acidic isopropanol and its quantity quantified spectrophotometrically at 570 nm (1420 Multilabelcounter VICTOR3, PerkinElmer, Waltham, MA, USA). Metabolic activity was expressed as relative to control (untreated cells incubated in HBSS).

### 3.15. In Vitro Scratch Wound Healing Assay

In vitro scratch wound healing assay was performed according to Blažević et al., 2016 [42]. The HaCaT cells were seeded onto 24-well plates at a density of 10^5^ cells/well and a volume of 500 µL/well and allowed to reach adequate confluence over 24 h in DMEM supplemented with 10% FBS and 5% antibiotic. Thereafter, the medium was removed and replaced with serum-free medium. After 24 h, a sterile 10 µL pipette tip was used to scrape across each well, creating a “wound” with a cell-free area. The cell monolayer was washed gently with HBSS (pH 6.0) to remove detached cells and cell debris. The wounds were exposed to the extracts’ solutions in HBSS for 2 h. Each well was marked below the plate surface to allow the identification of the same scratched area. After a 2 h treatment, the cells were washed with HBSS and incubated with serum-free medium in a volume of 500 µL/well. Wounds exposed to HBSS were used as a negative control. In vitro wound epithelization was monitored over 48 h, every 24 h, using phase-contrast microscopy (10× magnification; Primovert, Carl Zeiss AG, Oberkochen, Germany). The scratch area was measured using the ImageJ software (National Institutes of Health, Bethesda, MD, USA). The percentage of wound closure (PWC) was expressed as the percentage of scratch closure in relation to the initial scratch area, according to Equation (9):

(9) $$\mathrm{PWC}\;\left(\%\right)=\frac{A_{0}-A_{t}}{A_{0}}\times 100$$

where A_0_ is the scratch area at time 0 and A_t_ is the corresponding scratch area at 24 or 48 h.

### 3.16. Statistical Analysis

Design-Expert software v. 8.0.6 (Stat-Ease, Minneapolis, MN, USA) was used for the experimental design preparation (Box–Behnken) and validation (ANOVA) of Box–Behnken results. For evaluation of antioxidant and enzyme inhibiting activity, the results were presented as the mean ± standard deviation of three measurements. IC_50_ values were calculated using regression analysis. For wound healing assay, two independent experiments were performed, using three wells for each treatment. Statistical comparisons were made between the extracts using Students’ *t*-test (GraphPad Prism) and Dunnett’s post hoc test was used for comparison with the control. *p*-values < 0.05 were considered statistically significant.

## 4. Conclusions

*E. purpurea* aerial parts contain caffeic acid derivatives, potent cosmeceutical ingredients. In this work, the UAE method for preparation of *E. purpurea* bioactive extracts was developed. The extraction was performed using mixtures of water with glycerol, an environmentally friendly and safe solvent, used as a vehicle and active ingredient in cosmetic products. The extraction was optimized to obtain the extracts with the highest amount of phenolic acid and the best antiradical activity. The prepared extracts displayed excellent radical scavenging, Fe^2+^ chelating and antioxidant activity. In addition to that, collagenase, elastase and tyrosinase inhibitory activities, as well as their anti-inflammatory activity, indicate excellent antiaging properties of the extracts. The hyaluronidase inhibiting and wound healing effects were especially pronounced. The conducted research confirms a significant potential of *E. purpurea* extracts as valuable ingredients of cosmeceuticals with antiaging and wound healing properties.

## Figures and Tables

**Table 1:** Levels of independent variables in the Box–Behnken design, concentration of phenolic acids and IC_50_ value of the radical scavenging activity (RSA IC_50_) of the extracts.

Run	Std	X_1_	X_2_	X_3_	X_4_	Caf	Cic	TPA	RSA IC_50_
1	14	70	70	72	40	28.17	91.39	119.56	14.53
2	19	50	55	360	40	27.58	88.74	116.32	8.17
3	6	70	55	360	20	28.68	92.21	120.89	8.65
4	2	90	40	216	40	13.70	61.11	74.81	19.9
5	5	70	55	72	20	31.55	103.26	134.81	18.29
6	1	50	40	216	40	21.23	65.28	86.51	19.2
7	21	70	40	216	20	23.19	79.58	102.77	41.58
8	26	70	55	216	40	29.24	96.86	126.1	14.9
9	13	70	40	72	40	25.82	85.41	111.23	15.51
10	11	50	55	216	60	29.25	94.15	123.4	10.75
11	3	50	70	216	40	23.93	77.59	101.52	11.07
12	12	90	55	216	60	21.37	75.9	97.27	16.33
13	25	70	55	216	40	27.38	90.33	117.71	8.33
14	17	50	55	72	40	29.03	90.58	119.61	8.32
15	7	70	55	72	60	29.62	98.63	128.25	9.16
16	20	90	55	360	40	22.36	78.6	100.96	23.53
17	29	70	55	216	40	27.41	86.64	114.05	14.3
18	10	90	55	216	20	20.37	73.95	94.32	54.03
19	16	70	70	360	40	28.39	92.58	120.97	9.76
20	8	70	55	360	60	30.82	103.02	133.84	8.27
21	24	70	70	216	60	28.46	94.37	122.83	9.43
22	23	70	40	216	60	29.13	97.92	127.05	23.53
23	15	70	40	360	40	22.72	78.13	100.85	23.52
24	28	70	55	216	40	21.91	74.26	96.17	8.67
25	9	50	55	216	20	26.76	84.36	111.12	19.66
26	22	70	70	216	20	27.4	89.79	117.19	8.71
27	4	90	70	216	40	20.06	65.24	85.3	17.28
28	18	90	55	72	40	20.74	76.8	97.54	19.1
29	27	70	55	216	40	28.7	91.82	120.52	12.79

X_1_ = Glycerol concentration (%, *w*/*w*), X_2_ = Temperature (°C), X_3_ = Ultrasonication power (W), X_4_ = Time (min), Caf = Caftaric acid concentration (µg/mL), Cic = Cichoric acid concentration (µg/mL), TPA = Total phenolic acid concentration (µg/mL), RSA IC_50_ = Radical scavenging activity IC_50_ (µL extract/µL).

**Table 2:** Coefficients of the models’ polynomial equations (*a* × X_1_^2^ + *b* × X_2_^2^ + *c* × X_3_^2^ + *d* × X_4_^2^ + *e* × X_1_ × X_2_ + *f* × X_1_ × X_3_ + *g* × X_1_ × X_4_ + *h* × X_2_ × X_3_ + *i* × X_2_ × X_4_ + *j* × X_3_ × X_4_ + *k* × X_1_ + *l* × X_2_ + *m* × X_3_ + *n* × X_4_ + o) in terms of coded factors.

Response	Unit	The Equation Coefficients														
		*a*	*b*	*c*	*d*	*e*	*f*	*g*	*h*	*i*	*j*	*k*	*l*	*m*	*n*	*o*
Caf	µg/mL	−4.3 *	−2.4 *	1.8 *	1.9 *	0.9	0.8	−0.4	0.8	−1.2	1.0	−3.3 *	1.7 *	−0.4	0.9	26.9
Cic	µg/mL	−12.4 *	−6.6 *	6.0 *	7.0 *	−2.0	0.9	−2.0	2.1	−3.4	3.9	−5.8 *	3.6 *	−1.1	3.4	88.0
TPA	µg/mL	−18.4 *	−10.7 *	8.6 *	9.7 *	3.9	1.7	−2.3	2.9	−4.7	4.9	−10.7 *	7.0 *	−1.4	4.3	114.9
(RSA IC_50_)^−1/2^	µL extract/µL	0	0	0	0	0	0	0	0	0	0	0.7 *	−0.7 *	−0.1	−0.6 *	3.9

X_1_ = Glycerol concentration (%, *w*/*w*), X_2_ = Temperature (°C), X_3_ = Ultrasonication power (W), X_4_ = Time (min), Caf = Caftaric acid concentration (µg/mL), Cic = Cichoric acid concentration (µg/mL), TPA = Total phenolic acid concentration (µg/mL), RSA IC_50_ = Radical scavenging activity IC_50_ (µL extract/µL). * = Significant model terms.

**Table 3:** Analysis of variance (ANOVA) for the fitted quadratic models for optimization of *E. purpurea* extraction.

	Caf					Cic				
	*R*^2^ = 0.8958; *R*_a_^2^ = 0.79158; *R*_p_^2^ = 0.6933					*R*^2^ = 0.8765; *R*_a_^2^ = 0.7531; *R*_p_^2^ = 0.6634				
Source	SS	df	MS	*F* Value	*p*-value	SS	df	MS	*F* Value	*p*-value
Model	436.6	14	31.2	8.59	0.0001	4264.7	14	304.6	6.85	0.0005
LoF	16.7	10	1.6	0.20	0.9833	333.5	10	33.3	0.46	0.8535
PE	34.1	4	8.5			289.1	4	72.3		
	**TPA**					**IC_50_ RSA**				
	***R*^2^ = 0.8823; *R*_a_^2^ = 0.7647; *R*_p_^2^ = 0.5814**					***R*^2^ = 0.4928; *R*_a_^2^ = 0.4083; *R*_p_^2^ = 0.2382**				
Source	SS	df	MS	*F* Value	*p*-value	SS	df	MS	*F* Value	*p*-value
Model	7380.8	14	527.2	7.50	0.0003	15.9	4	4.0	5.83	0.0020
LoF	467. 9	10	46.8	0.36	0.9121	15.5	20	0.8	3.60	0.1111
PE	516.4	4	129.1			0.9	4	0.2		

SS = Sum of squares, df = Degrees of freedom, MS = Mean square, *r*_A_^2^ = Adjusted *r*^2^, *r*_P_^2^ = Predicted *r*^2^, LoF = Lack of fit, PE = Pure error, Caf = Caftaric acid concentration (µg/mL), Cic = Cichoric acid concentration (µg/mL), TPA = Total phenolic acid concentration (µg/mL), RSA IC_50_ = Radical scavenging activity IC_50_ (µL extract/µL).

**Table 4:** Predicted and observed values for the optimized extracts.

Extract	Measured Response	X_1_	X_2_	X_3_	X_3_	Resp_pred_	Resp_ms_	RD
		(%. *w*/*w*)	(°C)	(W)	(min)			(%)
TPA-opt	Caf	70	60	360	60	32.37	31.82	-1.7
TPA-opt	Cic	70	60	360	60	107.16	113.11	5.6
TPA-opt	TPA	70	60	360	60	139.53	144.93	3.9
RSA-opt	RSA IC_50_	50	70	144	55	4.90	5.32	8.6

X_1_ = Glycerol concentration (%, *w*/*w*), X_2_ = Temperature (°C), X_3_ = Ultrasonication power (W), X_4_ = time (min), Caf = Caftaric acid concentration (µg/mL), Cic = Cichoric acid concentration (µg/mL), TPA = Total phenolic acid concentration (µg/mL), RSA IC50 = Radical scavenging activity IC50 (µL extract/µL), Rsppred/ms–RD = Response deviation, calculated as (Rspms − Rsppred)/Rsppred × 100.

## Data Availability

Not applicable.

## References

[1] Pandey A., Jatana G.K., Sonthalia S. (2020). Cosmeceuticals. StatPearls.

[2] Costa R., Santos L. (2017). Delivery Systems for Cosmetics—From Manufacturing to the Skin of Natural Antioxidants. Powder Technol..

[3] de Lima Cherubim D.J., Martins C.V.B., Fariña L.O., da Silva de Lucca R.A. (2020). Polyphenols as Natural Antioxidants in Cosmetics Applications. J. Cosmet. Dermatol..

[4] Chemat F., Vian M.A., Cravotto G. (2012). Green Extraction of Natural Products: Concept and Principles. Int. J. Mol. Sci..

[5] Chemat F., Vian M.A., Ravi H.K., Khadhraoui B., Hilali S., Perino S., Tixier A.-S.F. (2019). Review of Alternative Solvents for Green Extraction of Food and Natural Products: Panorama, Principles, Applications and Prospects. Molecules.

[6] Wolfson A., Dlugy C., Shotland Y. (2007). Glycerol as a Green Solvent for High Product Yields and Selectivities. Environ. Chem. Lett..

[7] Karsch-Völk M., Barrett B., Kiefer D., Bauer R., Ardjomand-Woelkart K., Linde K. (2014). Echinacea for Preventing and Treating the Common Cold. Cochrane Database Syst. Rev..

[8] Echinaceae purpureae Herba. https://www.ema.europa.eu/en/medicines/herbal/echinaceae-purpureae-herba.

[9] Senica M., Mlinsek G., Veberic R., Mikulic-Petkovsek M. (2019). Which Plant Part of Purple Coneflower (*Echinacea purpurea* (L.) Moench) Should Be Used for Tea and Which for Tincture?. J. Med. Food.

[10] (2013). European Pharmacopoeia.

[11] Peng Y., Sun Q., Park Y. (2019). The Bioactive Effects of Chicoric Acid as a Functional Food Ingredient. J. Med. Food.

[12] Koriem K.M.M. (2020). Caftaric Acid: An Overview on Its Structure, Daily Consumption, Bioavailability and Pharmacological Effects. Biointerface Res. Appl. Chem..

[13] Yotsawimonwat S., Rattanadechsakul J., Rattanadechsakul P., Okonogi S. (2010). Skin Improvement and Stability of *Echinacea purpurea* Dermatological Formulations. Int. J. Cosmet. Sci..

[14] Riciputi Y., Diaz-de-Cerio E., Akyol H., Capanoglu E., Cerretani L., Caboni M.F., Verardo V. (2018). Establishment of Ultrasound-Assisted Extraction of Phenolic Compounds from Industrial Potato by-Products Using Response Surface Methodology. Food Chem..

[15] Fumić B., Jug M., Končić M.Z. (2019). Optimization of Ultrasound-Assisted Extraction of Phenolic Antioxidants from Lotus Corniculatus. Croat. Chem. Acta.

[16] Pandey A., Belwal T., Sekar K.C., Bhatt I.D., Rawal R.S. (2018). Optimization of Ultrasonic-Assisted Extraction (UAE) of Phenolics and Antioxidant Compounds from Rhizomes of *Rheum moorcroftianum* Using Response Surface Methodology (RSM). Ind. Crop. Prod..

[17] Momchev P., Ciganović P., Jug M., Marguí E., Jablan J., Končić M.Z. (2020). Comparison of Maceration and Ultrasonication for Green Extraction of Phenolic Acids from *Echinacea purpurea* Aerial Parts. Molecules.

[18] Spagnol C.M., Assis R.P., Brunetti I.L., Isaac V.L.B., Salgado H.R.N., Corrêa M.A. (2019). In Vitro Methods to Determine the Antioxidant Activity of Caffeic Acid. Spectrochim. Acta. A. Mol. Biomol. Spectrosc..

[19] Barel A., Paye M., Maibach H. (2001). Main Cosmetic Vehicles. Handbook of Cosmetic Science and Technology.

[20] Mlakar A., Batna A., Dudda A., Spiteller G. (1996). Iron (II) Ions Induced Oxidation of Ascorbic Acid and Glucose. Free Radic. Res..

[21] Ratz-Łyko A., Arct J. (2019). Resveratrol as an Active Ingredient for Cosmetic and Dermatological Applications: A Review. J. Cosmet. Laser Ther. Off. Publ. Eur. Soc. Laser Dermatol..

[22] Thiele J., Elsner P. (2001). Oxidants and Antioxidants in Cutaneous Biology.

[23] Zhu X., Huang F., Xiang X., Fan M., Chen T. (2018). Evaluation of the Potential of Chicoric Acid as a Natural Food Antioxidant. Exp. Ther. Med..

[24] Ganceviciene R., Liakou A.I., Theodoridis A., Makrantonaki E., Zouboulis C.C. (2012). Skin Anti-Aging Strategies. Dermato-Endocrinol..

[25] Aziz J., Shezali H., Radzi Z., Yahya N.A., Kassim N.H.A., Czernuszka J., Rahman M.T. (2016). Molecular Mechanisms of Stress-Responsive Changes in Collagen and Elastin Networks in Skin. Skin Pharmacol. Physiol..

[26] Uitto J. (1989). Connective Tissue Biochemistry of the Aging Dermis: Age-Associated Alterations in Collagen and Elastin. Clin. Geriatr. Med..

[27] Ersoy E., Ozkan E.E., Boga M., Yilmaz M.A., Mat A. (2019). Anti-Aging Potential and Anti-Tyrosinase Activity of Three Hypericum Species with Focus on Phytochemical Composition by LC–MS/MS. Ind. Crop. Prod..

[28] Imokawa G. (2008). Recent Advances in Characterizing Biological Mechanisms Underlying UV-Induced Wrinkles: A Pivotal Role of Fibrobrast-Derived Elastase. Arch. Dermatol. Res..

[29] Chaiyana W., Charoensup W., Sriyab S., Punyoyai C., Neimkhum W. (2021). Herbal Extracts as Potential Antioxidant, Anti-Aging, Anti-Inflammatory, and Whitening Cosmeceutical Ingredients. Chem. Biodivers..

[30] Wittenauer J., Mäckle S., Sußmann D., Schweiggert-Weisz U., Carle R. (2015). Inhibitory Effects of Polyphenols from Grape Pomace Extract on Collagenase and Elastase Activity. Fitoterapia.

[31] Papakonstantinou E., Roth M., Karakiulakis G. (2012). Hyaluronic Acid: A Key Molecule in Skin Aging. Dermato-Endocrinol..

[32] Chaiyana W., Anuchapreeda S., Punyoyai C., Neimkhum W., Lee K.-H., Lin W.-C., Lue S.-C., Viernstein H., Mueller M. (2019). *Ocimum sanctum* Linn. as a Natural Source of Skin Anti-Ageing Compounds. Ind. Crop. Prod..

[33] Facino R.M., Carini M., Aldini G., Marinello C., Arlandini E., Franzoi L., Colombo M., Pietta P., Mauri P. (1993). Direct Characterization of Caffeoyl Esters with Antihyaluronidase Activity in Crude Extracts from *Echinacea angustifolia* Roots by Fast Atom Bombardment Tandem Mass Spectrometry. Farm. Soc. Chim. Ital..

[34] Lengers I., Herrmann F., Le Borgne M., Jose J. (2020). Improved Surface Display of Human Hyal1 and Identification of Testosterone Propionate and Chicoric Acid as New Inhibitors. Pharmaceuticals.

[35] Pazyar N., Yaghoobi R., Rafiee E., Mehrabian A., Feily A. (2014). Skin Wound Healing and Phytomedicine: A Review. Skin Pharmacol. Physiol..

[36] Chang T.-S. (2009). An Updated Review of Tyrosinase Inhibitors. Int. J. Mol. Sci..

[37] Honisch C., Osto A., de Matos A.D., Vincenzi S., Ruzza P. (2020). Isolation of a Tyrosinase Inhibitor from Unripe Grapes Juice: A Spectrophotometric Study. Food Chem..

[38] Bessada S.M.F., Alves R.C., Oliveira M.B.P.P. (2018). Coffee Silverskin: A Review on Potential Cosmetic Applications. Cosmetics.

[39] Chandra S., Chatterjee P., Dey P., Bhattacharya S. (2012). Evaluation of in Vitro Anti-Inflammatory Activity of Coffee against the Denaturation of Protein. Asian Pac. J. Trop. Biomed..

[40] Penkova R., Goshev I., Gorinstein S., Nedkov P. (1999). Stability of Collagen during Denaturation. J. Protein Chem..

[41] Colombo I., Sangiovanni E., Maggio R., Mattozzi C., Zava S., Corbett Y., Fumagalli M., Carlino C., Corsetto P.A., Scaccabarozzi D. (2017). HaCaT Cells as a Reliable in Vitro Differentiation Model to Dissect the Inflammatory/Repair Response of Human Keratinocytes. Mediat. Inflamm..

[42] Blažević F., Milekić T., Romić M.D., Juretić M., Pepić I., Filipović-Grčić J., Lovrić J., Hafner A. (2016). Nanoparticle-Mediated Interplay of Chitosan and Melatonin for Improved Wound Epithelialisation. Carbohydr. Polym..

[43] Kawano Y., Patrulea V., Sublet E., Borchard G., Iyoda T., Kageyama R., Morita A., Seino S., Yoshida H., Jordan O. (2021). Wound Healing Promotion by Hyaluronic Acid: Effect of Molecular Weight on Gene Expression and in Vivo Wound Closure. Pharmaceuticals.

[44] Ibezim E.C., Momoh M.A., Onyishi V.I., Nwabunike I., Odimegwu D.C., Ibezim N.E., Nzekwe N.J. (2018). Evaluating the Ethyl-Acetate Fraction of Crude Methanol Leaf Extract of Ocimum Gratissimum Formulated as Ointments for Wound Healing Properties Using the Excision Wound Model. J. Pharm. Allied Sci..

[45] Jug M., Končić M.Z., Kosalec I. (2014). Modulation of Antioxidant, Chelating and Antimicrobial Activity of Poplar Chemo-Type Propolis by Extraction Procures. LWT-Food Sci. Technol..

[46] Končić M.Z., Barbarić M., Perković I., Zorc B. (2011). Antiradical, Chelating and Antioxidant Activities of Hydroxamic Acids and Hydroxyureas. Molecules.

[47] Rajić Z., Končić M., Miloloža K., Perković I., Butula I., Bucar F., Zorc B. (2010). Primaquine-NSAID Twin Drugs: Synthesis, Radical Scavenging, Antioxidant and Fe^2+^ Chelating Activity. Acta Pharm..

[48] Zhang Y., Fu Y., Zhou S., Kang L., Li C. (2013). A Straightforward Ninhydrin-Based Method for Collagenase Activity and Inhibitor Screening of Collagenase Using Spectrophotometry. Anal. Biochem..

[49] Jabłonowska M., Ciganović P., Jablan J., Marguí E., Tomczyk M., Končić M.Z. (2021). Silybum Marianum Glycerol Extraction for the Preparation of High-Value Anti-Ageing Extracts. Ind. Crop. Prod..

[50] Jiratchayamaethasakul C., Ding Y., Hwang O., Im S.-T., Jang Y., Myung S.-W., Lee J.M., Kim H.-S., Ko S.-C., Lee S.-H. (2020). In Vitro Screening of Elastase, Collagenase, Hyaluronidase, and Tyrosinase Inhibitory and Antioxidant Activities of 22 Halophyte Plant Extracts for Novel Cosmeceuticals. Fish. Aquat. Sci..

